# Severe Dysphagia is Rare After Magnetic Sphincter Augmentation

**DOI:** 10.1007/s00268-022-06573-2

**Published:** 2022-04-29

**Authors:** Milena Bologheanu, Aleksa Matic, Joy Feka, Reza Asari, Razvan Bologheanu, Franz M. Riegler, Lisa Gensthaler, Bogdan Osmokrovic, Sebastian F. Schoppmann

**Affiliations:** 1 Department of Surgery, Division of General Surgery, Medical University of Vienna, Waehringer Guertel 18-20, 1090 Vienna, Austria; 2 Department of Anaesthesia, Intensive Care Medicine and Pain Medicine, Medical University of Vienna, Waehringer Guertel 18-20, 1090 Vienna, Austria; 3 Reflux Ordination, Mariannengasse 10/4/9, 1090 Vienna, Austria; 4 Department of Trauma Surgery, Krankenhaus Oberwart, Dornburggasse 80, 7400 Oberwart, Austria

## Abstract

**Background:**

Dysphagia remains the most significant concern after anti-reflux surgery, including magnetic sphincter augmentation (MSA). The aim of this study was to evaluate postoperative dysphagia rates, its risk factors, and management after MSA.

**Methods:**

From a prospectively collected database of all 357 patients that underwent MSA at our institution, a total of 268 patients were included in our retrospective study. Postoperative dysphagia score, gastrointestinal symptoms, proton pump inhibitor intake, GERD-HRQL, Alimentary Satisfaction, and serial contrast swallow imaging were evaluated within standardized follow-up appointments. To determine patients’ characteristics and surgical factors associated with postoperative dysphagia, a multivariable logistic regression analysis was performed.

**Results:**

At a median follow-up of 23 months, none of the patients presented with severe dysphagia, defined as the inability to swallow solids or/and liquids. 1% of the patients underwent endoscopic dilatation, and 1% had been treated conservatively for dysphagia. 2% of the patients needed re-operation, most commonly due to recurrent hiatal hernia. Two patients underwent device removal due to unspecific discomfort and pain. No migration of the device or erosion by the device was seen. The LINX® device size ≤ 13 was found to be the only factor associated with postoperative dysphagia (OR 5.90 (95% CI 1.4–24.8)). The postoperative total GERD-HRQL score was significantly lower than preoperative total score (2 vs. 19; *p* = 0.001), and daily heartburn, regurgitations, and respiratory complains improved in 228/241 (95%), 131/138 (95%) and 92/97 (95%) of patients, respectively.

**Conclusions:**

Dysphagia requiring endoscopic or surgical intervention was rare after MSA in a large case series. LINX® devices with a size < 13 were shown to be an independent risk factor for developing postoperative dysphagia.

## Introduction

Gastroesophageal reflux disease (GERD) affects up to 28% of the western population [1–4]. Although surgical treatment has been proven to be safe and effective in reflux control [1, 5–7], the rates of laparoscopic Nissen fundoplication (LNF), the current standard in anti-reflux surgery [8–11], have decreased in the last decade [12–15]. This decline can be explained by the feared side effects and long-term failure [12–15]. To reduce this therapy gap and provide alternative surgical options, new less invasive procedures such as magnetic sphincter augmentation (MSA) have been introduced.

MSA has been shown to be as safe and effective as LNF, but with lower rates of postoperative gas-bloating syndrome and inability to belch and/or vomit in studies so far [2, 16–20]. The rate of postoperative dysphagia varies with the follow-up: Acute postoperative dysphagia is common and affects approximately 30% of all patients, while long-term dysphagia rates drop to 7% after 3 years [15, 21–24]. A possible explanation lies in the different postoperative diet of MSA patients: unlike LNF patients who ingest a semi-liquid diet for at least 2 weeks postoperatively, MSA patients are advised to eat solid foods to prevent a restrictive fibrous capsule from building around the device [24]. Additionally, postoperative dietary regimes and the management differ among centers [2, 9, 17, 25, 26], thus making treating postoperative dysphagia challenging and underlining the importance of further studies evaluating this issue.

The aim of this study is to evaluate the postoperative outcome, especially dysphagia rates, its risk factors and management, in GERD patients undergoing MSA in our high-volume specialized reflux center.

## Materials and methods

From a prospective database including a total of 357 patients, that underwent MSA for GERD in a period of 10 years (2012–2022) in our specialized reflux center in Vienna, we included a total number of 268 patients (173 male and 95 female), that underwent MSA in the period from 2012 to 2020 with a minimum of 2 months follow-up in retrospective data analyses.

### Preoperative assessment

All patients underwent a standardized interview, clinical examination, upper GI endoscopy, and esophageal functioning tests (EFT): high-resolution manometry and 24-h-Impedance-pH-metry. MSA was indicated in patients with diagnosed GERD by positive pH results or increased total reflux episodes with positive symptom correlation on EFT, esophagitis on endoscopy, or typical GERD symptoms sensitive to PPI medication, who did not have spastic or absent esophageal motility on the preoperative EFT.

All operations were performed by or assisted by the same surgeon, who is the head/chief of the surgical upper GI unit in our university hospital with 24 years of surgical experience and 15 years of experience in upper GI surgery, in a standardized manner. Since 2014, hiatoplasty has been performed in all patients in principle. These procedures were conducted without esophageal bougie by hiatal dissections and crural closures with 2–5 stitches using non-absorbable sutures.

### Magnetic sphincter augmentation

MSA was performed as previously described [19]: briefly after the mobilization of the esophagogastric junction, the adequate ring size was measured with the sizing tool and the magnetic ring was wrapped around the lower end of the lower esophageal sphincter.

### Sizing of the LINX® Device

The MSA device was sized without foreign bodies using the designated tool while avoiding tension or compression of the esophagus and was closed until it popped off. The sizing tool was wiggled to prevent squeezing the esophagus. If measurement yielded 10–12 beads, 3 beads would be added. If measurement yielded 13, we would add 3 or 2, depending on whether squeezing with the sizing tool occurred*.* If measurement yielded 14 or 15, we would add 2, if 17 beads were available. If measurement yielded 16, we would use this size if no squeezing occurred*.* If measurement yielded 16 and squeezing occurred, the procedure would not be performed.

### Postoperative care

Postoperatively, all patients received an unrestricted diet, putting an emphasis on regular intake of a small portion of solid foods every 2 h while awake for 5 weeks, to avoid the development of dysphagia due to formation of scar tissue surrounding the device. On the first postoperative day, a contrast swallow with Iopamidole was performed in all patients. After at least one overnight stay, patients were discharged from the hospital if the contrast swallow study was unremarkable.

### Postoperative assessment

The median follow-up time was 23 months (IQR 9–36). It was performed by the same surgical resident, who was very rarely, only occasionally and by chance assigned to the procedure as the second assistant during her residency training rotations. A standardized in-person interview that assessed postoperative gastrointestinal symptoms—presence and frequency of heartburn, regurgitations and any preoperative symptoms, presence and frequency of dysphagia, PPI intake, and GERD-Health-related-Quality-of-Life score (GERD-HRQL) was used. The frequency and severity of postoperative dysphagia was assessed using the classification of Saeed et al., where the ability to swallow can be scored from 0 for inability to swallow to 5 for normal swallowing [27].

Postoperative complications were assessed using the Clavien–Dindo classification [28]. Further hospital readmissions, emergency or elective re-operations were documented. In selected patients with recurrent symptoms, upper GI endoscopy and EFTs were performed.

### Statistical analysis

Statistical analysis was performed using SPSS® statistics 20.0 (IBM, Armonk, NY), the Python scientific ecosystem and the statsmodels module [29, 30]. The data were described using median (interquartile range) or mean (range). Statistical analysis appropriate for non-parametric data was used. Categorical variables were assessed using the Fisher exact test and continuous data using the Wilcoxon Rank test as appropriate. A multivariable logistic regression of the preoperative clinical and objective parameters predictive of postoperative dysphagia risk was performed. Feature selection using the Akaike information criterion (AIC) was performed to optimize the logistic regression model. The results of the logistic regression models are presented as coefficient and standard error, odds ratios (OR), 95% confidence intervals, and *p*-values. Statistical significance was defined as a *p*-value < 0.05.

This study (2293/2017) was approved by the Institutional Review Board of the Medical University of Vienna, Austria. In order to secure data security, all patient-related data are collected in a password-secured database. The database is secured on the server of the Institute of Surgery.

## Results

In our cohort, a total number of 268 (173 male and 95 female) that underwent MSA in the period from 2012 to 2020 with a minimum of 2 months follow-up were included in the analysis. Demographics and preoperative findings of all 268 individuals are shown in Table 1.

**Table 1:** Demographic data and results of preoperative diagnostics of all patients

	Patients
Total	*n* = 268 (100%)
Sex (M vs. F)	173 vs. 95
Median Age (IQR)	50 (38–59)
Median BMI (IQR)	25.5 (23–28.3)
HH^a^ present HH > 3 cm	210 (78%) 62 (23%)
Median Total pH < 4% (IQR)	5.4% (2.7–10.6)
Median Total Reflux episodes (IQR)	63 (43–84)
Median LES^b^ resting pressure (IQR)	17.8 mmHg (11.07–25.07)
Median IRP^c^ (IQR)	9 mmHg (6–12)
Presence of IEM^d^	18 (7%)
Presence of BE^e^	30 (11%)
Use of PPIs	235 (88%)
Presence of heartburn	241 (90%)
Presence of regurgitation	138 (52%)
Presence of respiratory symptoms	97 (36%)

Data were obtained and statistics applied, as described in Methods

^a^Hiatal hernia, ^b^Lower esophageal sphincter, ^c^Integrated relaxation pressure, ^d^Ineffective esophageal motility, ^e^Barrett’s Esophagus

### Preoperative symptoms

The most common preoperative symptoms were heartburn, regurgitations, and respiratory symptoms. Regarding preoperative dysphagia, 96% of patients reported absolutely no difficulty swallowing solids or liquids, while 1% of patients rarely had difficulties swallowing solids only, and 3% of the patients had occasional difficulties swallowing solids.

### Surgery

The median operating room time was 30 min (IQR 25–34, range 9–95). The surgical approach was laparoscopic in all patients. No perioperative complications were seen. The median MSA-Device size implanted was 15 (IQR 14–15, range 12–16). 81% of patients received additional crural closure. The median hospital stay was 1 day (IQR 1–2, range 1–7). None of the patients showed a complete contrast stop at the gastroesophageal junction in the first postoperative contrast swallows performed.

### Postoperative symptom control

The median follow-up time was 23 months (IQR 9–36). Daily bothersome heartburn, regurgitations and respiratory symptoms were improved in 95% (*n* = 228/241), 95% (*n* = 131/138) and 95% (*n* = 92/97) of the patients, while full elimination of heartburn, regurgitation and respiratory symptoms was seen in 80% (*n* = 191/241), 76% (*n* = 105/138) and 72% (*n* = 70/97) of the patients, respectively. Patients with residual symptoms seeking further surgical expertise underwent postoperative EFTs: from all the patients with residual regurgitations (*n* = 33), only 4 had a postoperative EFT, where 2 showed normal findings and 2 showed increased acid exposure. From all patients with residual heartburn (*n* = 50), only 13 underwent postoperative EFT with 9 showing no abnormalities and 4 showing an increased acid exposure.

### Postoperative dysphagia

**Fig. 1:**
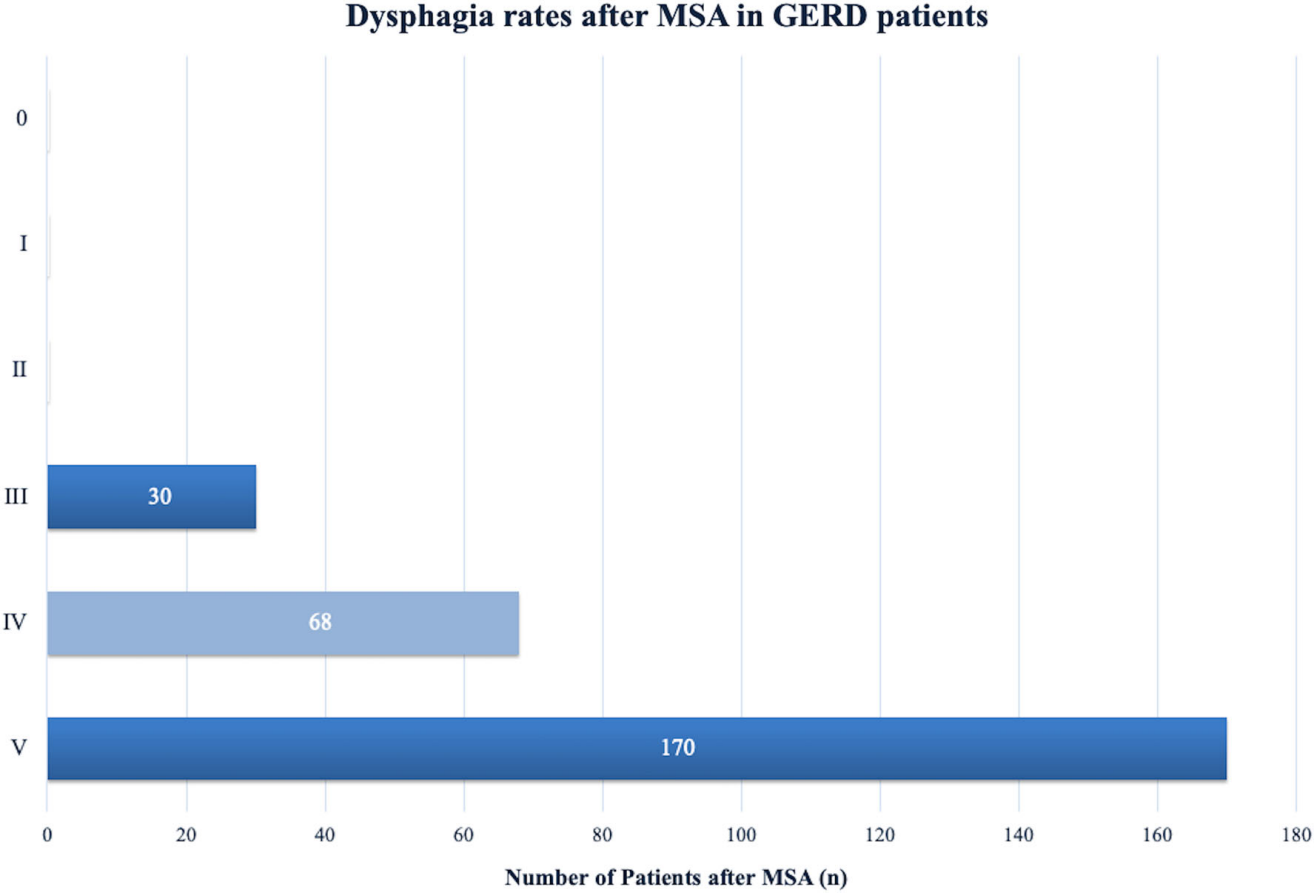
Dysphagia rates after MSA in GERD patients

At follow-up, a total of 63% (*n* = 170/268) of individuals reported absolutely no difficulty swallowing with solids or liquids after MSA. Rarely difficulties swallowing solids only were reported by 25% (*n* = 68/268) of the patients, while 11% (*n* = 30/268) patients had occasional difficulties swallowing solids. Finally, severe dysphagia, defined as not being able to swallow solids or/and liquids, was not seen in any of the patients at follow-up. These findings are presented in Fig. 1.

Three (1%) of our patients underwent endoscopic balloon dilatation due to dysphagia. One patient underwent endoscopic dilatation, followed by a course of oral corticosteroids (5 mg for 5 days, followed by 2.5 mg for the next 5 days) 2 months after the operation, after which the dysphagia was resolved. The second patient reported only being able to swallow liquids 1 month after MSA and underwent endoscopic dilatation, however with minimal improvement. The contrast swallow and HRM showed no abnormalities, so the patient underwent two courses of oral corticosteroids (a year apart) and was told to follow our strict food intake regime for the next month. At follow-up, the patient reported improvement of the dysphagia with occasional difficulties swallowing solids. The last patient underwent endoscopic dilatation due to difficulty swallowing solids twice—2 weeks after MSA with minimal improvement and 2 years later with complete resolution of the dysphagia.

Two (1%) patients underwent conservative oral corticosteroid treatment for postoperative dysphagia. One patient reported occasional difficulty swallowing solids with dyspepsia after every swallow 8 months after MSA. The contrast swallow showed no abnormalities, while the HRM showed in 6/10 swallows a spasm. He then underwent a course of oral corticosteroids, after which his dysphagia fully resolved. The other patient reported not being able to swallow solids 6 weeks after MSA. The contrast swallow also showed no abnormalities, and she underwent a course of oral corticosteroids, after which she reported only rarely difficulties swallowing solids.

### Preoperative predictors of postoperative dysphagia

We analyzed 15 possible preoperative predictors in a multivariable logistic regression analysis: age > 60, sex, overweight, obesity, IEM, Barrett’s esophagus (BE), hiatal hernia, hiatal hernia >3 cm, esophagitis, dysphagia, IRP >15 mmHg, LES resting pressure >43 mmHg, LINX® size < 14, LINX® size <13 and follow-up time <12 months, as demonstrated in Table 2 and Table 3 and found LINX® device size ≤ 13 to be the only predictor associated with postoperative dysphagia defined as either “occasional difficulties swallowing solids” or the need for endoscopic dilatation or corticosteroid therapy after MSA (OR 5.90 (95% CI 1.4–24.8)). After variable selection using the AIC, a LINX® device size ≤ 13 remained the only predictor of postoperative dysphagia in the new multivariable analysis. We selected the input features from the available data based on our experience and published data: postoperative dysphagia, ineffective swallows, and the absence of paraesophageal hernia have been previously identified as risk factors for postoperative dysphagia [24]. Further, as another study found age <40 to be an independent predictor of a long-term successful postoperative outcome [31], we added age with a higher cutoff >60 as a potential factor for postoperative dysphagia. LINX® size was chosen as multiple studies show it is advisable to oversize the MSA device to prevent dysphagia and device removal [24, 31, 32] and a follow-up <12 months due to the fact that dysphagia has been shown to be higher in the acute postoperative phase, while long-term dysphagia rates drop to 7% after 3 years [15, 21–24]. Lastly, the rest of the factors like sex, BMI, esophagitis and BE were selected out of curiosity and availability of the data.

**Table 2:** Potential preoperative predictors for postoperative dysphagia according to the multivariable logistic model

	Coef	SE	*z*	*P* >|*z*|	Odds ratio	95% Confidence interval
Age > 60	−0.22	0.45	0.50	0.62	0.80	0.33–1.93
Sex	0.67	0.40	1.70	0.09	1.96	0.90–4.25
Overweight	−1.14	0.39	2.91	0.00	0.32	0.15–0.69
Obesity	−0.03	0.63	0.04	0.97	0.97	0.28–3.35
IEM^a^	−0.74	0.82	0.90	0.37	0.48	0.10–2.38
BE^b^	0.25	0.55	0.46	0.65	1.29	0.44–3.78
HH^c^	−1.27	0.37	3.48	0.00	0.28	0.13–0.57
HH > 3 cm	0.23	0.50	0.47	0.64	1.26	0.48–3.35
Esophagitis	−0.81	0.42	1.95	0.05	0.45	0.20–1.00
Preoperative dysphagia	0.51	0.77	0.67	0.51	1.67	0.37–7.47
IRP^d^ ≥ 15 mmHg	0.17	0.51	0.34	0.73	1.19	0.43–3.26
LES^e^ resting pressure ≥ 43 mmHg	−0.17	1.27	0.13	0.89	0.85	0.07–10.10
LINX® size ≤ 14	−0.92	0.51	1.83	0.07	0.40	0.15–1.06
LINX® size ≤ 13	**1.78**	**0.73**	**2.42**	**0.02**	**5.90**	**1.40–24.80**
Follow-up time < 12 months	0.22	0.40	−0.56	0.58	0.80	0.36–1.74

Data were obtained and statistics applied, as described in Methods

^a^Ineffective esophageal motility, ^b^Barett’s esophagus, ^c^Hiatal hernia, ^d^Integrated relaxation pressure, ^e^Lower esophageal sphincter

**Table 3:** Potential preoperative predictors for postoperative dysphagia according to the multivariate logistic model after a variable selection using AIC

	Coef	SE	*Z*	*P* >|*z*|	Odd ratio	95% Confidence interval
Sex	0.5734	0.3561	1.6104	0.1073	1.77437	0.882977–3.565678
HH	−1.2587	0.2928	−4.2996	0.0000	0.284019	0.160014–0.504124
Esophagitis	−0.8507	0.4050	−2.1004	0.0357	0.427110	0.193099–0.944711
Overweight	−1.1108	0.3304	−3.3617	0.0008	0.329287	0.172308–0.629280
LINX® size ≤ 14	−0.8831	0.4769	−1.8519	0.0640	0.413495	0.162392–1.052874
LINX® size ≤ 13	**1.8711**	**0.7035**	**2.6597**	**0.0078**	**6.495119**	**1.636024–25.786035**

Data were obtained and statistics applied, as described in Methods

^a^Hiatal hernia

### Postoperative adverse effects and surgical revisions

According to the Clavien–Dindo classification, we observed 9 (3%) patients with a postoperative complication grade III [28]. As mentioned above, 3 patients needed endoscopic dilatation due to dysphagia. Further 6 (2%) of the patients required re-operation. Two of the patients underwent an explant of the MSA device: One patient due to abdominal trauma and epigastric discomfort 14 months after the MSA, while the other patient reported spastic retrosternal pain during swallowing, undergoing the explant 8 days after the MSA. The other 4 patients required revision hiatoplasty due to the occurrence of a paraesophageal HH after 6 months, 1 year, 1 year and 2 years, respectively. No erosion or migration of the device was observed. Postoperative outcomes are represented in Table 4.

**Table 4:** Postoperative outcome including postoperative symptoms, adverse effects, reoperative rates and patients satisfaction after magnetic sphincter augmentation (MSA)

	Patients
Total	*n* = 268 (100%)
Persistent dysphagia	0
Endoscopic dilatation	3 (1%)
Reoperation	6 (2%)
Device removal	2 (1%)
Re-Hiatoplasty	4 (1.5%)
Gas bloating syndrom	16 (6%)
Ability to belch/vomit	222 (83%)
Median total GERD-HRQL score (IQR)	2 (IQR 0–4)
Median AS^a^ (IQR)	9/10 (IQR 8–10)
Willing to undergo surgery again	214 (80%)
Patient overall satisfied	232 (87%)
Use of PPIs	25 (9%)

Data were obtained and statistics applied, as described in Methods

^a^Alimentary satisfaction

### Quality of life

Prior to surgery, 50% of patients had completed the GERD-HRQL score. The preoperative median total GERD-HRQL was 19 (IQR 13–26), while the postoperative median total GERD-HRQL was 2 (IQR 0–4), showing a significant reduction in the GERD-HRQL total score (19 vs. 2, *p* = 0.001). Quality of Life results are presented in Table 4.

## Discussion

In this study, we analyzed postoperative outcomes, particularly dysphagia rates, its risk factors and management after MSA. We found no patients with severe dysphagia, defined as not being able to swallow solids and/or liquids, and only 11% of patients reported occasional difficulties swallowing solids at follow-up. Moreover, only 1% of the patients underwent endoscopic dilatation, unlike previous studies, that found endoscopic dilatation rates ranging from 2.4 to 31% [24, 25, 31, 33]. This difference could be explained by diverse postoperative dietary regimes, the intraoperative measurement protocol of the LINX® device, and management of postoperative dysphagia. In our cohort, all patients underwent an unrestricted food diet, with solid food intake every 2 h while awake for the first 5 weeks starting on the day of the surgery. If patients presented with dysphagia within 8 weeks after the primary operation, further dietary training with intake of small food bites every hour while awake for the next month is advised according to our protocol. Additionally, they receive a course of oral corticosteroids starting with 5 mg of Prednisolone once daily for 5 days, followed by 2.5 mg of Prednisolone for the next 5 days. This regime was then adapted to 25 mg twice daily for 5 days, followed by 5 mg twice daily for the next 5 days. The dietary training prevents the formation of a fibrous capsule around the closed ring, while concomitant low-dose corticosteroid therapy reduces the systemic inflammation and tissue scarring. If no improvement occurs, endoscopic pneumatic dilatation with a 20 mm balloon would be recommended, followed by a 10-day course of low-dose corticosteroids. Our dysphagia management changed with time: in 2013 we performed an endoscopic dilatation in 2 patients within 2 and 4 weeks, respectively, with minimal improvement, after which we stopped performing pneumatic dilatation before 8 weeks postoperatively. Also, based on clinical experience and expert opinion, we implemented low-dose oral corticosteroid therapy and increased the dosage with time, as patients tolerated the medication well, without developing systemic side effects, while achieving the desired effect.

Currently, it is unclear which patients are at higher risk of developing long-term dysphagia or poor postoperative outcome after MSA. A retrospective review in 2020 found three independent predictors of long-term dysphagia: preoperative dysphagia, less than 80% effective swallows in the preoperative HRM, and the absence of a large or paraesophageal hernia [24]. Likewise, a study following 124 patients 6–12 years after MSA found that age less than 40 is an independent predictor of a long-term successful postoperative outcome [31]. In our study, we found that implanting the LINX® device size ≤ 13 is associated with occasional and severe dysphagia (OR 5.90 (95% CI 1.4–24.8)). This finding is consistent with previous studies, as multiple studies show it is advisable to oversize the MSA device to prevent dysphagia and device removal [24, 31, 32]. Similarly, in our practice determining the appropriate LINX® device size is crucial—using the sizing tool we close it around the esophagus, gradually tightening it until it pops open. Depending was measurement it yields, we add 1–3 beads, oversizing the MSA device as recommended.

In our cohort 2% of the patients underwent surgical revision: 4 due to recurrent HH and 2 due to non-specific pain and discomfort in the chest area. This result is also on the lower end compared to previous publications. Furthermore, the cause of the re-operation also differs. None of our patients needed device removal due to dysphagia or reoccurrence of GERD symptoms, which was described as the most common cause found in the safety analysis of the first 1000 patients after MSA in 2015 [33]. More recently, a long-term study in 2020 reported that 9.4% of patients needed device removal in the course of 12 years. However, the most common cause in the first 6 years is erosion of the device and regurgitations [31].

The median follow-up time in our study was 23 months, with the longest follow-up being 102 months, 8.5 years after MSA. As previously shown, the LINX® device was effective in GERD symptom control. Improvement of daily bothersome heartburn, regurgitations and respiratory symptoms was seen 95% of the patients each, and only 9% of the individuals reported a need for use of PPIs postoperatively [2, 17, 18, 31, 34]. Furthermore, we observed a significant reduction in the median GERD HRQL total score (19 vs. 2, *p* = 0.001) after MSA showing a significant improvement in GERD-related quality of life. This finding is also in line with studies so far, all proving that MSA significantly improves patient satisfaction and quality of life [2, 17, 18, 31, 34].

Finally, we acknowledge certain limitations of our study inherent to retrospective design, further missing standardized reporting criteria, although we used the applicable STROBE checklist criteria. Further, lack of a control group and different follow-up time between our patients, due to the effort to increase our study population and diversity should be taken into consideration. Lastly, there was a lack of objective (EFTs) assessment of GERD elimination postoperatively, thus relying purely on subjective patient evaluation of outcomes. However, due to the invasiveness of the procedure and the majority of patients being asymptomatic, we had ethical considerations, as well as logistic difficulties to indicate the necessity of and organize such a procedure in our large cohort. Further prospective, larger studies assessing risk factors for developing and the optimal management of postoperative dysphagia, as well as objective postoperative testing, are needed.

## Conclusion

Severe dysphagia, defined as not being able to swallow solids or/and liquids, requiring further endoscopic or surgical intervention was rare after MSA in our cohort, when patients received the appropriate postoperative dietary regime, endoscopic dilatation before the first 8 weeks was avoided, and larger LINX® device sizes were implanted.
